# The correlational study of the 24 solar terms and meteorological factors with the acute exacerbation of bipolar disorder

**DOI:** 10.1016/j.cpnec.2025.100336

**Published:** 2026-01-09

**Authors:** Jian Chen, Tingting Wu, Hongyu Wu, Jingying Zhou, Wenfei Li

**Affiliations:** a School of Mental Health and Psychological Science, Anhui Medical University, Hefei, China; b Affiliated Psychological Hospital of Anhui Medical University, Hefei, China; c Anhui Provincial Mental Health Center, Hefei, China; d Hefei Fourth People's Hospital, Hefei, China

**Keywords:** Bipolar disorder, 24 solar terms, Meteorological factors, Weather changes, Acute episode

## Abstract

**Background:**

Bipolar disorder (BD) is a severe mood disorder, and increasing evidence suggests that acute episodes of BD may exhibit seasonal patterns. However, the relationship between acute BD episodes and meteorological factors remains a contentious issue in academia. This study aims to investigate the distribution of BD acute episodes across 24 solar terms, offering a fresh perspective on the link between BD acute episodes and meteorological factors.

**Methods:**

This analysis was based on retrospectively collected hospitalization records from patients with acute BD episodes at Anhui Mental Health Center (2020–2022), and contemporaneous meteorological data.

**Results:**

The two peaks of BD manic episodes occurred during Rainwater and Grain in Ear solar terms, whereas the peak of depressive episodes was observed during Rainwater and Summer Solstice solar terms. BD manic episodes were significantly correlated with temperature differences, interday temperature differences and interday wind speed differences. Depressive episodes were significantly correlated with the temperature difference, interday wind speed difference and atmospheric pressure. Linear regression analysis revealed that the interday atmospheric pressure difference and interday temperature difference were significantly associated with acute BD episodes.

**Conclusion:**

This study conducted a large-scale survey on the distribution of acute BD episodes across 24 solar terms in the Chinese population and their correlations with meteorological factors and sociodemographic characteristics. The findings indicate that the distribution of BD acute episodes varies across the 24 solar terms and that there is a correlation between BD acute episodes and certain meteorological factors, particularly drastic changes in temperature and atmospheric pressure, which may account for the differences in the number of BD acute episodes across the solar terms.

## Highlights

• Significantly positively correlated with temperature differences and inter-day wind speed variations.
• Atmospheric pressure shows a significant negative correlation with depressive episodes.
• Inter-day pressure and temperature differences are positive predictors of acute episodes.
• Marital status is significantly associated with episode distribution across solar terms.
• Supports the development of personalized prevention strategies using meteorological and solar term characteristics.

## 1 Introduction

Bipolar disorder (BD) is a severe mood disorder characterized by extreme mood swings, encompassing both depressive and manic episodes [1]. In recent years, a growing body of evidence has suggested that acute episodes of BD might follow seasonal patterns [2,3], garnering significant attention from researchers and clinicians alike. However, there is still no general agreement within the academic community on the relationships between acute BD episodes and meteorological factors. While some studies have indicated that higher temperatures and increased sunlight exposure are associated with an elevated risk of manic episodes, and lower temperatures and reduced daylight are linked to more depressive episodes [4]; [5], others have reported no significant link between daily meteorological patterns and these episodes [6,7]. Additionally, research has suggested that sudden weather changes could impact physiological and psychological states [8], potentially correlating with acute episodes of BD. For example, meteorological factors such as seasonal variations in daylight, temperature fluctuations, and humidity changes might affect biological rhythms, neurotransmitter levels, and hormonal levels [9,10], thereby influencing mood regulatory mechanisms and triggering acute episodes of BD [11]. However, research in this area is relatively scarce, and the findings are inconsistent.

The 24 solar terms constitute a significant concept in traditional Chinese culture that reflects patterns of meteorological and seasonal changes, dividing the year into 24 periods [12], each associated with specific climatic characteristics and natural phenomena. Given the close relationship between the 24 solar terms and meteorological changes [13], we hypothesize that they might be correlated with acute episodes of BD. Moreover, different populations may exhibit varying sensitivities to meteorological and weather changes [14], which could be related to the seasonal patterns of acute BD episodes.

The primary objective of this study is to investigate the distribution of acute BD episodes across the 24 solar terms and determine the relationship between these episodes and specific meteorological factors (including temperature, atmospheric pressure, wind speed, precipitation, humidity, and sunlight exposure) as well as their sudden changes (defined as interday differences in temperature, atmospheric pressure, wind speed, and sunlight duration).

By collecting and analyzing the clinical data of BD patients, meteorological data, and sociodemographic information, this study employs multivariate statistical analysis methods to explore potential connections between acute BD episodes and meteorological factors as well as sociodemographic characteristics. We hope that this research will provide a fresh perspective on the seasonal characteristics of BD and offer a scientific basis for clinicians to develop personalized prevention and treatment strategies.

## 2 Methods

### 2.1 Research design

This study utilized retrospectively collected clinical data from patients with BD hospitalized at the Anhui Mental Health Center (2020–2022) and contemporaneous meteorological data, aiming to explore the meteorological connections between the 24 solar terms and acute BD episodes.

### 2.2 Research subjects

The study included patients who were hospitalized due to acute episodes of bipolar disorder (BD) between 2020 and 2022. All patients were diagnosed with BD according to the Diagnostic and Statistical Manual of Mental Disorders, Fifth Edition (DSM-V) and were currently experiencing an acute manic or depressive episode requiring hospitalization; patients with unclear diagnoses and those with mixed episodes were excluded.

### 2.3 Data collection

#### 2.3.1 Patient data

Patient data were extracted from the electronic medical records of Anhui Mental Health Center, including the number of BD manic and depressive episodes across different solar terms, as well as the sociodemographic characteristics of the patients (sex, marital status, occupational status, and age; see Table 1). The data collected refer to the current hospitalization episode for each patient.

**Table 1:** General information of all bipolar disorder acute episode patients.

		Number	Percentage (%)			Number	Percentage (%)
Gender	Male	1770	52.60 %	Marital Status	Married	1554	46.18 %
	Female	1595	48.20 %		Unmarried	1488	44.22 %
					Others	323	9.60 %
Age	≤18	163	4.84 %				
	19–35	1605	47.70 %	Occupation	Unemployed	1082	32.15 %
	36–59	1257	37.36 %		Student	364	10.82 %
	≥60	340	10.10 %		Others	1919	57.03 %

#### 2.3.2 Meteorological data

Meteorological data were obtained from the Anhui Meteorological Center, a regional authoritative meteorological data provider with high reliability and completeness of data. The collected meteorological data included temperature, temperature difference, atmospheric pressure, precipitation, humidity, wind speed, and sunlight exposure corresponding to the 24 solar terms during 2020–2022. All meteorological variables were averaged by solar term to match the temporal scale of BD acute episode data. Additionally, we calculated the meteorological variables that reflect sudden weather changes: interday temperature difference (the difference between the highest and lowest temperatures of two consecutive days), interday atmospheric pressure difference (the difference in average atmospheric pressure between two consecutive days), interday wind speed difference (the difference in the highest wind speed between two consecutive days), and interday sunlight difference (the difference in sunlight duration between two consecutive days). These variables were derived from the original daily meteorological data and averaged by solar term.

### Ethical approval

This study was approved by the Institutional Review Committee of the Fourth People's Hospital of Hefei (Approval No: HSY-IRB-PJ-LWF(HFSY2022YB06)).

### 2.4 Statistical analysis

All data entry and analysis were performed via SPSS software (version 27.0). The normality and homogeneity of variance of the data were assessed through the Kolmogorov‒Smirnov test and Levene's test. Nonparametric statistical methods were employed for data that did not conform to a normal distribution. A p value less than 0.05 was considered statistically significant.

- First, we evaluated the differences in the number of BD acute manic and depressive episodes across different solar terms.
- Second, Pearson correlation analysis was used to assess the linear correlation between different meteorological factors (averaged by solar term) and acute BD onset.
- Before establishing the linear regression model, multicollinearity diagnosis was conducted (variance inflation factor [VIF] < 3 was considered no significant multicollinearity) to exclude highly correlated variables, ensuring model stability. A total of 11 meteorological variables were included in the correlation analysis, and 5 key variables were selected for linear regression analysis based on theoretical relevance and correlation results. The rationale for model choice was to identify the most impactful meteorological factors while controlling for multicollinearity.
- Bonferroni correction was used to adjust for multiple comparisons, with the adjusted p value < 0.05 considered statistically significant.
- Finally, chi-square tests were used to analyze whether there were statistically significant differences between sociodemographic characteristics and the number of BD acute-onset episodes across different solar terms.

## 3 Results

### 3.1 Basic information of included patients

A total of 3365 patients with acute BD episodes were included in this study, including 1770 males (52.60 %) and 1595 females (48.20 %). The detailed sociodemographic characteristics are shown in Table 1.

### 3.2 Seasonal peaks of BD acute episodes

**Fig. 1:**
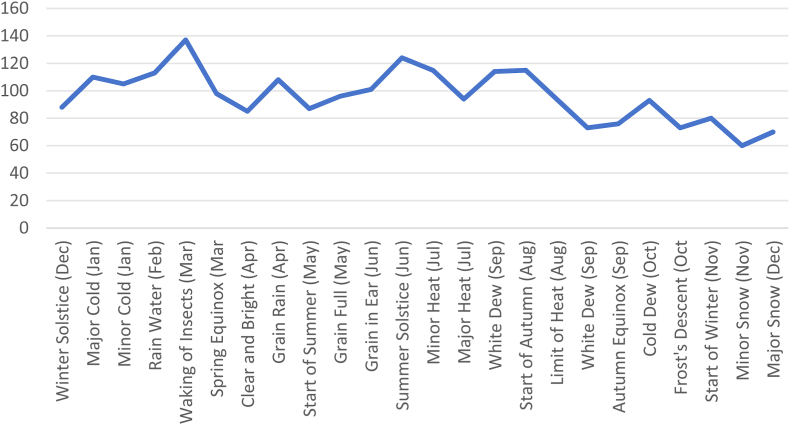
Distribution of the number of Bipolar Manic Eoisodes across the 24 Solar Terms.

**Fig. 2:**
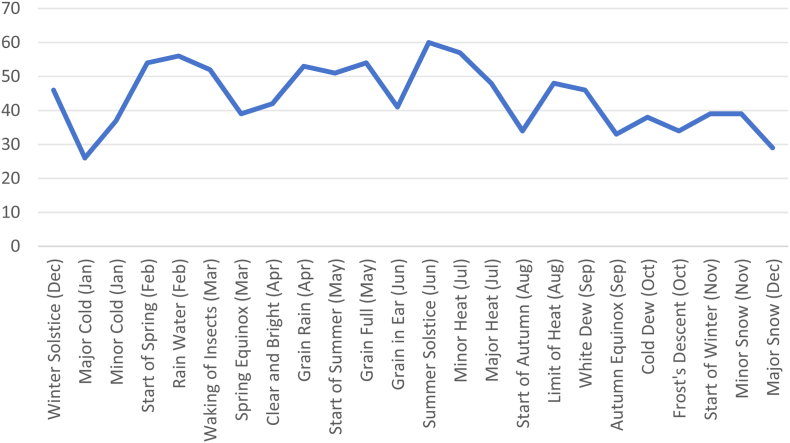
Distribution of the number of Bipolar Depressive Episodes across the 24 Solar Terms.

The results of this study indicate (Fig. 1, Fig. 2; note: the corresponding months for each solar term are marked in the figure legends for easier interpretation) that the manic episodes of BD peak during the solar terms of Rainwater (February) and Grain in Ear (June), whereas depressive episodes peak during the solar terms of Rainwater (February) and Summer Solstice (June). Moreover, the number of manic and depressive episodes in spring (March–May) and summer (June–August) significantly exceeds those in autumn (September–November) and winter (December–February). This finding reveals a potential link between acute BD episodes and specific solar terms.

### 3.3 Pearson correlation analysis

According to the results of this study (Table 2), BD manic episodes are significantly positively correlated with temperature differences, interday temperature differences and interday wind speed differences (r = 0.237, 0.256, 0.273, p < 0.05 after Bonferroni correction). BD depressive episodes are significantly positively correlated with temperature differences and interday wind speed differences (r = 0.281, 0.269, p < 0.05 after Bonferroni correction), whereas BD depressive episodes are significantly negatively correlated with atmospheric pressure (r = −0.261, p < 0.05 after Bonferroni correction). It should be noted that these correlation coefficients (r ∼0.24–0.28) are relatively low, indicating a weak-to-moderate strength of association, which suggests that meteorological factors are only one part of the complex multifactorial mechanisms underlying acute BD episodes.

**Table 2:** Correlation analysis of various meteorological factors with BD manic and depressive episodes.

	Manic Episode Correlation Coefficient	p value	Depressive Episode Correlation Coefficient	p value
Temperature	0.097	0.418	0.190	0.110
Temperature Difference	0.237∗	0.045	0.281∗	0.017
Atmospheric Pressure	−0.184	0.122	−0.261∗	0.027
Precipitation	−0.086	0.475	0.034	0.777
Humidity	−0.062	0.606	0.029	0.810
Wind Speed	−0.068	0.571	0.046	0.700
Sunlight	0.078	0.514	0.038	0.749
interday temperature difference	0.256∗	0.030	0.213	0.072
interday atmospheric pressure difference	0.159	0.182	0.083	0.489
interday wind speed difference	0.273∗	0.020	0.269∗	0.022
interday sunlight difference	0.136	0.256	0.157	0.188

∗*p* < 0.05 ∗∗*p* < 0.01.

### 3.4 Linear regression analysis

According to the study results (Table 3, Table 4), BD manic episodes: interday atmospheric pressure difference (β = 0.422, p < 0.01 after Bonferroni correction) and interday temperature difference (β = 0.367, p < 0.05 after Bonferroni correction) were significantly associated with the number of manic episodes, whereas atmospheric pressure (β = −0.609, p < 0.01 after Bonferroni correction) was negatively associated with manic episodes.

**Table 3:** Linear regression analysis results of BD manic episodes with various meteorological factors.

	Nonstandardized Coefficients		Standardized Coefficients	t	*p*	Collinearity Diagnosis	
	*B*	Standard Error	*Beta*			VIF	Tolerance
Constant	−27.331	22.079	–	−1.238	0.220	–	–
Humidity	0.420	0.224	0.263	1.880	0.065	1.848	0.541
Atmospheric Pressure	−0.072	0.017	−0.609	−4.183	0.000∗∗	2.004	0.499
interday atmospheric pressure difference	0.423	0.148	0.453	2.866	0.006∗∗	2.368	0.422
interday wind speed difference	0.437	0.348	0.150	1.257	0.213	1.353	0.739
interday temperature difference	0.137	0.058	0.367	2.369	0.021∗	2.269	0.441
*R*^2^	0.303						
*Adjusted R*^2^	0.250						
*F*	*F* (5,66) = 5.734,*p* = 0.000						
D-W Value	1.368						

Dependent Variable: Number of Manic Episodes.

∗*p* < 0.05 ∗∗*p* < 0.01.

**Table 4:** Linear regression analysis results of BD depressive episodes with various meteorological factors.

	Nonstandardized Coefficients		Standardized Coefficients	t	*p*	Collinearity Diagnosis	
	*B*	Standard Error	*Beta*			VIF	Tolerance
Constant	−25.677	11.463	–	−2.240	0.028∗	–	–
Humidity	0.321	0.116	0.371	2.768	0.007∗∗	1.848	0.541
Atmospheric Pressure	−0.043	0.009	−0.663	−4.744	0.000∗∗	2.004	0.499
interday atmospheric pressure difference	0.213	0.077	0.422	2.779	0.007∗∗	2.368	0.422
interday wind speed difference	0.286	0.181	0.182	1.583	0.118	1.353	0.739
interday temperature difference	0.084	0.030	0.418	2.809	0.007∗∗	2.269	0.441
*R*^2^	0.357						
*Adjusted R*^2^	0.308						
*F*	*F* (5,66) = 7.325,*p* = 0.000						
D-W Value	2.116						
Dependent Variable: Number of Depressive Episodes							

∗*p* < 0.05 ∗∗*p* < 0.01.

BD depressive episodes: humidity (β = 0.371, p < 0.01 after Bonferroni correction), interday atmospheric pressure difference (β = 0.422, p < 0.01 after Bonferroni correction), and interday temperature difference (β = 0.418, p < 0.01 after Bonferroni correction) were significantly associated with the number of depressive episodes, whereas atmospheric pressure (β = −0.663, p < 0.01 after Bonferroni correction) was negatively associated with depressive episodes.

**Fig. 3:**
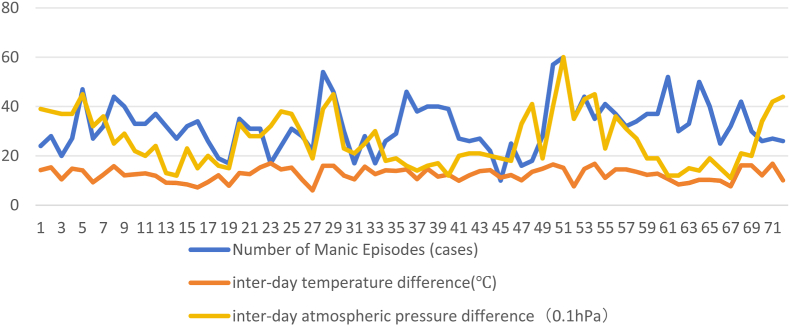
Trends of the number of manic episodes and changes in temperature and pressure differences for each solar term from 2020 to 2022.

**Fig. 4:**
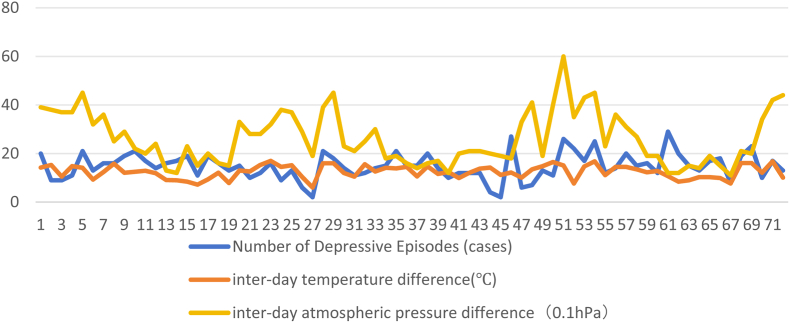
Trends of the number of depressive episodes and changes in temperature and pressure differences for each solar term from 2020 to 2022.

Furthermore, trend charts (Fig. 3, Fig. 4; note: the data in the trend charts are consistent with the 2020–2022 data collection period) show that interday temperature difference and interday atmospheric pressure difference are associated with acute episodes of BD.

### 3.5 Chi-square test

According to the study results (Table 5), marital status (χ^2^ = 81.313, p < 0.01 after Bonferroni correction) is significantly associated with the number of BD acute episodes across different solar terms, whereas sex (χ^2^ = 29.916, p > 0.05 after Bonferroni correction), age (χ^2^ = 68.637, p > 0.05 after Bonferroni correction) and occupation (χ^2^ = 53.038, p > 0.05 after Bonferroni correction) are not significantly associated with the number of BD acute episodes across different solar terms.

**Table 5:**
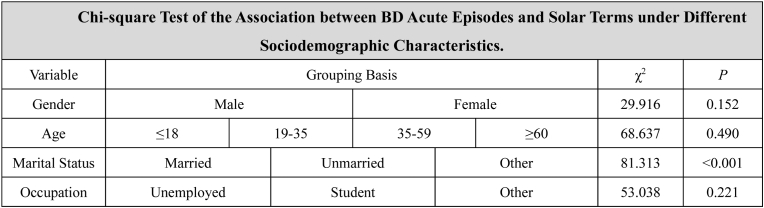
Chi-square test of the association between BD acute episodes and solar terms under different sociodemographic Characteristics.

## 4 Discussion

### 4.1 Seasonal patterns of acute BD episodes

Our study revealed that acute episodes of BD exhibit significant seasonal peaks at specific solar terms. The solar terms of Rainwater (February) in spring and Grain in Ear (June) in summer are closely associated with the peaks of manic episodes, whereas Rainwater (February) in spring and Summer Solstice (June) in summer coincide with the peak periods of depressive episodes. These solar terms are concentrated in spring and summer, which is consistent with previous research [15,16].

The solar terms in spring, such as Rainwater, not only mark revival and growth in nature but also lead to significant fluctuations in temperature and atmospheric pressure. These fluctuations may affect mood regulation by impacting human biological rhythms and neurotransmitter levels [17]. Additionally, the extended daylight hours and changes in natural colors in spring may have positive effects on mood, but they may also increase individuals' sensitivity to environmental changes, leading to instability in emotional states [18].

High temperatures and low atmospheric pressure in summer may be associated with mood states by affecting the body's thermoregulatory mechanisms and oxygen supply [19]. Longer daylight hours can increase sun exposure and enhance the synthesis of vitamin D and serotonin in the body, but excessive sunlight and high temperatures may also induce physiological stress responses, which are correlated with emotional stability [20]. Increased humidity and decreased sleep quality could also be significant factors related to mood regulation [21]; [22].

### 4.2 Correlations between meteorological factors and BD acute episodes

The most significant finding of our research is the substantial positive correlation between meteorological factors such as temperature and air pressure fluctuations and acute episodes of BD. This link may explain why acute episodes of BD vary across different seasons. Temperature fluctuations can not only directly affect the thermoregulatory system of the human body but also further disrupt the balance of key neurotransmitters in the brain, such as serotonin and dopamine [23]; [24], which are crucial for emotional stability and cognitive function [25]; [26]. In addition, drastic changes in temperature may indirectly be related to emotional stability by reducing people's health maintenance behaviors, such as regular exercise and adequate sleep [21]. Changes in air pressure, especially the negative correlation between air pressure and depressive episodes of BD, suggest that air pressure stability may be positively correlated with emotional regulation. This could be related to the effects of air pressure changes on brain blood flow and oxygen supply, which are associated with emotional states [27]. Moreover, people's discomfort with air pressure changes, such as discomfort in low-pressure environments, may also be correlated with emotional stability [28]. Changes in wind speed may also be associated with emotions, with drastic changes in wind speed being related to weather instability, which may increase individuals' sense of uncertainty and anxiety levels [29].

Notably, although we have provided some explanations for the correlation between weather fluctuations and acute episodes of BD, we currently lack a deeper understanding of the specific pathways. Furthermore, the relatively weak correlation coefficients (r ∼0.24–0.28) between meteorological factors and BD acute episodes indicate that these environmental factors are not the sole determinants. Acute BD episodes are likely the result of complex interactions among genetic predispositions, psychological factors, social environments, and meteorological conditions. For instance, genetic variations related to neurotransmitter metabolism may modulate an individual's sensitivity to weather changes, while chronic psychological stress could amplify the correlation between meteorological fluctuations and mood regulation. Therefore, the findings of this study should be interpreted within the context of multifactorial causality, and future research should integrate multiple dimensions to comprehensively explore the mechanisms underlying BD acute episodes.

### 4.3 Role of sociodemographic characteristics

Our study also revealed that different marital statuses are associated with differences in the number of acute episodes of BD across various solar terms. It is important to note that this association does not imply direct social causality, as unmeasured confounders (such as employment status, social isolation, and comorbidities) may also contribute to the observed differences. Married individuals may have a more stable social support system, which helps alleviate life stress, whereas unmarried, divorced, and widowed individuals may face more emotional challenges and social barriers [30]. The lack or insufficiency of social support may increase the risk of emotional fluctuations for these individuals, especially when dealing with life stress and psychophysiological stress [31]. Consequently, when faced with drastic weather changes, emotional fluctuations may become more pronounced, leading to a higher susceptibility to acute episodes of BD [32].

To further elaborate on this mechanism, we speculate that social support, as a key protective factor, may act as a “buffer” against the physiological stress induced by rapid weather changes. Married individuals often have more opportunities for emotional communication and practical assistance from their spouses, which can help regulate stress responses triggered by meteorological fluctuations (e.g., changes in cortisol levels and sympathetic nervous system activity). In contrast, individuals with insufficient social support may lack effective stress-coping resources, making their mood regulation systems more vulnerable to the impact of environmental changes such as temperature and air pressure fluctuations. For example, during the Rainwater solar term, when temperature and air pressure fluctuate drastically, married individuals may better adapt to these changes through spousal companionship and emotional support, thereby reducing the risk of acute BD episodes. This hypothesis is supported by previous studies indicating that social support can moderate the relationship between environmental stressors and mental health outcomes [31]; [32]. Future research could verify this buffering effect through longitudinal studies and physiological indicator measurements (e.g., stress hormone levels, heart rate variability) to provide more direct evidence for the underlying mechanism.

These findings indicate that sociodemographic characteristics also play a role in acute episodes of BD. Therefore, clinical intervention strategies should also consider patients’ background information. For example, providing social work services and psychological counseling for patients facing a lack of social support can be beneficial.

### 4.4 Significance and application prospects of the study

The results of this study hold significant implications for understanding the correlates of BD episodes. By identifying meteorological and demographic correlates of acute BD episodes, clinicians can develop more precise prevention and management strategies. For example, doctors could advise patients to monitor specific weather conditions and take preventive medication or increase the frequency of psychological monitoring during high-risk periods. Additionally, understanding patients' sociodemographic backgrounds, such as a lack of social and family support, can help doctors assess patients' sensitivity to environmental changes and provide appropriate psychological support and interventions. Finally, these findings also provide data support for public health policymakers in formulating policies to address the impact of weather changes on mental health.

### 4.5 Study limitations and future research directions

Although this study reveals significant correlations between meteorological factors and acute episodes of BD and highlights the role of sociodemographic characteristics, we acknowledge certain limitations. First, the number of hospital admissions cannot fully substitute for the number of acute episodes of bipolar disorder. Second, while we observed that certain meteorological factors (e.g., temperature and atmospheric pressure fluctuations) may be related to acute BD episodes, this study did not fully elucidate the specific mechanisms. Third, the study may have selection bias because the data are limited to a specific region and time period. Fourth, unmeasured confounding factors (e.g., individual physiological characteristics, genetic factors, and lifestyle) may influence acute episodes of BD. Fifth, the temporal alignment between acute episodes and solar term assignment is a potential limitation: episodes were categorized by hospital admission date, but the exact onset and duration of exacerbations prior to admission were unrecorded, which may lead to minor bias due to inconsistent correspondence between actual episode periods and assigned solar terms.

In light of these limitations, we propose the following directions for future research: (1) Elucidating the mechanism of action: conducting physiological and psychological studies to understand how meteorological factors affect human biological rhythms, neurotransmitter levels, hormone levels, and the immune system, thereby influencing mood states. (2) Considering individual differences: investigating the differences in sensitivity to meteorological changes among individuals and exploring the connections between these differences and genetics, lifestyle habits, and health conditions. (3) Comprehensive environmental factor analysis: expanding the scope of research to include other environmental factors (e.g., geographical environment and urbanization level) to fully assess their impact on acute BD episodes. (4) Developing personalized interventions: based on a deeper understanding of the mechanisms by which meteorological factors affect health, developing personalized prevention and intervention measures to reduce the risk of acute episodes in BD patients. (5) Promoting interdisciplinary research: encouraging cooperation among researchers from different disciplines (e.g., psychology, meteorology, biology, and medicine) to explore the relationships between meteorological factors and BD and promote the development of integrated solutions. (6) Prospective studies: conducting long-term follow-up studies to monitor the mood changes and environmental exposures of BD patients, precisely recording the exact onset and duration of acute episodes to achieve more accurate alignment with solar terms, to verify the findings of this study and further clarify the relationships between meteorological factors and acute BD episodes.

Through research in these directions, we hope to provide a more scientific basis for the prevention and treatment of BD, ultimately improving patients' quality of life and treatment outcomes.

## 5 Conclusions

In summary, our study revealed that the number of BD acute episodes varies across the 24 solar terms, and further analysis suggests that this variation may be related to drastic fluctuations in temperature and atmospheric pressure. Additionally, a lack of social support may increase individuals' sensitivity to weather changes. Understanding these differences can aid in clinical intervention and personalized prevention and control, providing more precise prevention and management measures. However, this study may have selection bias and unmeasured confounding factors, and future research needs to further explore how meteorological factors specifically act on the human body and affect mood regulatory mechanisms, consider individual differences, and develop personalized intervention measures.

## CRediT authorship contribution statement

**Jian Chen:** Writing – review & editing, Writing – original draft, Formal analysis, Data curation. **Tingting Wu:** Writing – original draft, Formal analysis, Data curation. **Hongyu Wu:** Formal analysis, Data curation. **Jingying Zhou:** Writing – review & editing. **Wenfei Li:** Funding acquisition.

## Ethical standards

This study was approved by the Institutional Review Committee of the Fourth People's Hospital of Hefei (Approval No: HSY-IRB-PJ-LWF(HFSY2022YB06)).

## Role of funding source

This work was supported by grants from the Scientific Research Promotion Plan of Anhui Medical University (2022xkjT005), the Clinical Applied Research Fund of the Fourth People's Hospital of Hefei City, Anhui Province (HFSY2022YB06).

## Declaration of competing interest

The authors declare no conflict of interest.

## Data Availability

Data supporting the findings of this study are available from the corresponding authors upon reasonable request.
